# The Ndr/LATS Kinase Cbk1 Regulates a Specific Subset of Ace2 Functions and Suppresses the Hypha-to-Yeast Transition in Candida albicans

**DOI:** 10.1128/mBio.01900-20

**Published:** 2020-08-18

**Authors:** Rohan S. Wakade, Laura C. Ristow, Mark A. Stamnes, Anuj Kumar, Damian J. Krysan

**Affiliations:** a Department of Pediatrics, Carver College of Medicine, University of Iowa, Iowa City, Iowa, USA; b Department of Molecular Physiology and Biophysics, Carver College of Medicine, University of Iowa, Iowa City, Iowa, USA; c Department of Molecular, Cellular, and Developmental Biology, University of Michigan, Ann Arbor, Michigan, USA; d Department of Microbiology/Immunology, Carver College of Medicine, University of Iowa, Iowa City, Iowa, USA; University of British Columbia

**Keywords:** *Candida albicans*, RAM pathway, hyphal morphogenesis

## Abstract

The regulation of Ace2 and morphogenesis (RAM) pathway is an important regulatory network in the human fungal pathogen Candida albicans. The RAM pathway’s two most well-studied components, the NDR/Lats kinase Cbk1 and its putative substrate, the transcription factor Ace2, have a wide range of phenotypes and functions. It is not clear, however, which of these functions are specifically due to the phosphorylation of Ace2 by Cbk1. To address this question, we first compared the transcriptional profiles of *CBK1* and *ACE2* deletion mutants. This analysis indicates that, of the large number of genes whose expression is affected by deletion of *CBK1* and *ACE2*, only 5.5% of those genes are concordantly regulated. Our data also suggest that Ace2 directly or indirectly represses a large set of genes during hyphal morphogenesis. Second, we generated strains containing *ACE2* alleles with alanine mutations at the Cbk1 phosphorylation sites. Phenotypic and transcriptional analysis of these *ace2* mutants indicates that, as in Saccharomyces cerevisiae, Cbk1 regulation is important for daughter cell localization of Ace2 and cell separation during yeast-phase growth. In contrast, Cbk1 phosphorylation of Ace2 plays a minor role in C. albicans yeast-to-hypha transition. We have, however, discovered a new function for the Cbk1-Ace2 axis. Specifically, Cbk1 phosphorylation of Ace2 prevents the hypha-to-yeast transition. To our knowledge, this is one of the first regulators of the C. albicans hypha-to-yeast transition to be described. Finally, we present an integrated model for the role of Cbk1 in the regulation of hyphal morphogenesis in C. albicans.

## Abstract (precis)

The regulation of Ace2 and morphogenesis (RAM) pathway is a key regulatory network that plays a role in many aspects of C. albicans pathobiology. In addition to characterizing the transcriptional effects of this pathway, we discovered that Cbk1 and Ace2, a key RAM pathway regulator-effector pair, mediate a specific set of the overall functions of the RAM pathway. We have also discovered a new function for the Cbk1-Ace2 axis: suppression of the hypha-to-yeast transition. Very few regulators of this transition have been described, and our data indicate that maintenance of hyphal morphogenesis requires suppression of yeast phase growth by Cbk1-regulated Ace2.

## INTRODUCTION

Candida albicans is one of the most common causes of human fungal infections, the severity of which ranges from life-threatening invasive disease to relatively minor but still consequential mucosal infections (1). As a component of the human mycobiome, C. albicans primarily colonizes the human oral cavity, gastrointestinal tract, and urogenital tract (2). Accordingly, it is well adapted to niches that differ greatly in their environmental characteristics (3). Regardless of the specific niche or site of infection, however, the ability of C. albicans to cause disease has been closely linked to its ability to undergo morphogenic transitions to either pseudohyphae or true hyphae (4). Consequently, the molecular mechanisms by which these transitions are regulated have been of keen interest to mycologists (5). Over the years, many signaling pathways have been shown to participate in this regulation, including the protein kinase A pathway, mitogen-activated protein kinase (MAPK) signaling pathways, and the regulation of Ace2 and morphogenesis (RAM) pathway (6). The RAM pathway is distinguished from other more extensively studied pathways by its apparent ability to modulate multiple transcriptional regulators (7). Here, we describe a set of experiments designed to identify the subset of Ace2 functions that are directly dependent on the RAM pathway and its kinase Cbk1.

The RAM pathway is conserved within fungi, including human pathogens, and its components regulate a wide variety of functions in these organisms (7). The effector components of the RAM pathway are an Ndr/LATS kinase such as Cbk1 in Saccharomyces cerevisiae and C. albicans and the zinc finger transcription factor Ace2 (7, 8). The activity of the kinase is dependent on a network of cofactors, including Mob2, Kic1, Hym1, Tao3, and Sog2. The RAM pathway was initially described in the model yeast S. cerevisiae, and its function during the cell cycle of this budding yeast has been extensively studied (8). Based on these studies, the function of Ace2 as a daughter cell-specific regulator (9) of cell separation gene expression that is dependent upon phosphorylation by Cbk1 has been firmly established (10). Cbk1 phosphorylates Ace2 within the nuclear export sequence which blocks export of Ace2 from daughter cell nuclei (10). In the absence of Cbk1 phosphorylation, Ace2 localization is disrupted, leading to defects in cell separation. The phosphorylation site motif for Cbk1 has been characterized biochemically and genetically leading to the following consensus sequences: HXRXXS/T and HRXXS/T (10). The histidine residue is critical for recognition, and substrates are not phosphorylated in its absence. In S. cerevisiae, the RNA binding protein Ssd1 is the other well-characterized Cbk1 substrate (11–13), and Cbk1 phosphorylation of Ssd1 plays an important role in the regulation of translation of specific sets of genes.

In contrast, the functions of the RAM pathway and the transcription factor Ace2 in the human fungal pathogen C. albicans have been examined more broadly (7). In addition to their role in polarized growth and cell separation (14, 15), the RAM pathway and Ace2 affect hyphal formation (14–16), response to hypoxia (16, 17), biofilm formation (17–19), adherence to abiotic surfaces (18), susceptibility to antifungal drugs (20), cell wall structure, including β-glucan masking (21, 22), and regulation of metabolic genes (16). Recently, alternative splicing has been implicated as an additional source of functional diversity for Ace2 in that one isoform appears to function as a transcription factor while the other is localized to the plasma membrane and has a role in septum dynamics (23). A genetic interaction screen reported by our group found that Cbk1 interacts with a wide range of functionally distinct genes, including those noted above as well as genes required for oxidative stress tolerance and nitrogen utilization (24). In a mouse model of disseminated candidiasis, MacCallum et al. found a very slight defect in survival for an *ace2*ΔΔ strain relative to that of the wild type (WT) (25). To circumvent the complicating factors of cell separation defects, our group used heterozygous mutants of *ACE2* and *CBK1* to show that both are haploinsufficient with respect to kidney burden early in the infection process (26). In summary, the RAM pathway directly or indirectly regulates a wide range of functions important to the biology and pathobiology of C. albicans.

One of the distinguishing features of the RAM pathway in C. albicans is that it appears to regulate multiple transcription factors. To date, three putative C. albicans Cbk1 substrates have been confirmed genetically or biochemically: Bcr1, Fkh2, and Ssd1. Gutiérrez-Escribano et al. showed that Cbk1-mediated phosphorylation of Bcr1 is important for its full function during biofilm formation (27), and Greig et al. demonstrated that Cbk1 phosphorylation of Fkh2 is important for hyphal morphogenesis (28). Finally, Lee et al. reported that mutants of Ssd1 lacking Cbk1 phosphosites failed to degrade *NRG1* mRNA during hyphal morphogenesis, providing a mechanism for the profound inability of *cbk1*ΔΔ mutants to generate hyphae (29).

In contrast to S. cerevisiae, Ace2 has not been experimentally confirmed to be a Cbk1 substrate in C. albicans. A large-scale phosphoproteomic analysis of C. albicans during hyphal formation showed that two of the three putative Cbk1 phosphorylation sites contain phosphates under those conditions (30). Although there is little doubt that Ace2 is regulated by Cbk1, we were interested in identifying Cbk1-dependent functions of Ace2. Here, we describe the generation of strains with the Cbk1 phosphorylation sites mutated to nonphosphorylatable alanine residues (*ace2*-2A and *ace2-*3A). As described below, our observations indicate that although both Cbk1 and Ace2 have a wide range of functions important to the biology of C. albicans, a very specific set of these functions is directly regulated by the phosphorylation of Ace2 by Cbk1, including maintaining a balance between hyphal and yeast transcriptional programs (31).

## RESULTS

### Cbk1 regulates a relatively small proportion of Ace2-dependent genes during yeast-phase growth.

The RAM pathway and Ace2 have been the subject of previous transcriptional profiling experiments using microarray technology. Mulhern et al. profiled *ace2*ΔΔ mutants during both yeast-phase growth and serum-induced hyphal growth (16). In addition, Song et al. examined the effect of deleting *MOB2*, the Cbk1 binding partner and activator, under similar conditions (20). Since RNA sequencing had not been used in either instance, we decided to compare the transcriptional profiles of *ace2*ΔΔ and *cbk1*ΔΔ mutants using this technique. We were particularly interested in identifying the set of genes that is regulated by both Cbk1 and Ace2.

**FIG 1:**
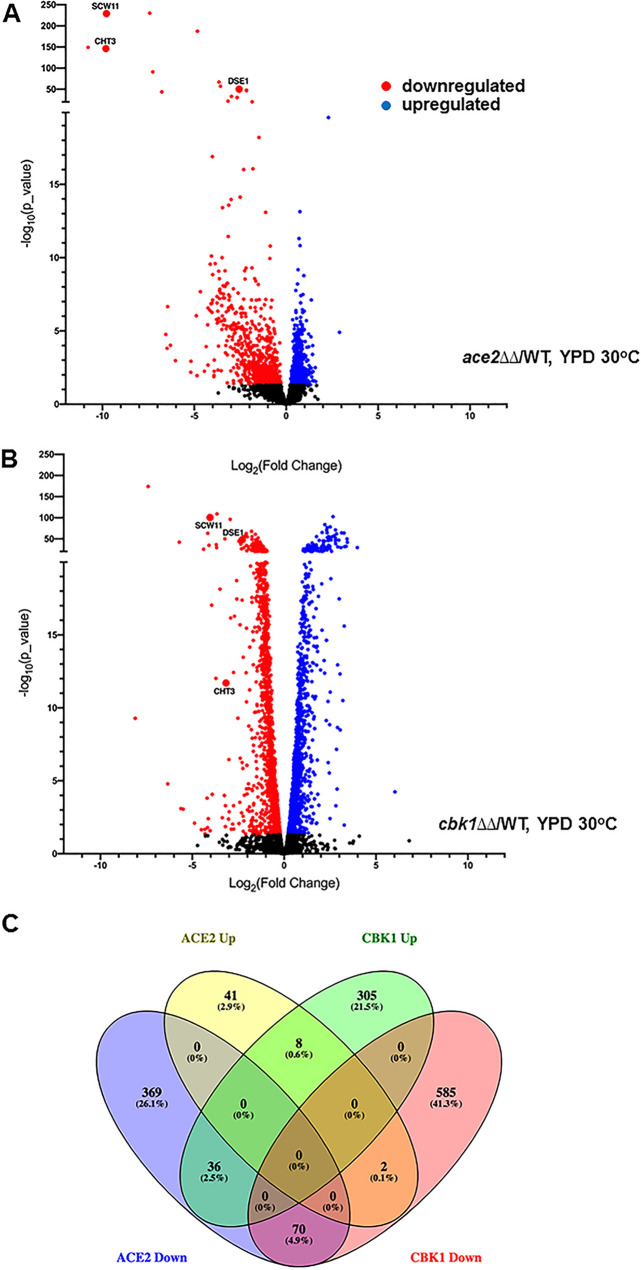
Ace2 and Cbk1 affect the expression of large sets of genes during yeast-phase growth, but only a fraction of the genes are regulated in common. Volcano plots of the gene expression profiles of *ace2*ΔΔ (A) and *cbk1*ΔΔ (B) mutants relative to that in the wild type under exponential growth in YPD at 30°C, with genes showing statistically significant (adjusted *P < *0.05) changes in expression. (C) Venn diagram of differentially expressed genes (+/−log_2_ 1 and *P* < 0.05) for *ace2*ΔΔ and *cbk1*ΔΔ mutants.

During yeast-phase growth at 30°C in rich yeast-peptone-dextrose (YPD), the expression of 526 genes was altered by at least 2-fold (adjusted *P* < 0.05) (full set of genes listed in Table S3 in the supplemental material) in the *ace2*ΔΔ mutant (Fig. 1A). The total number of genes affected by deletion of *ACE2* observed in our data set is very similar to that reported by Mulhern et al. (16) (363 with a false-discovery rate [FDR] of 0.23%; 645 with an FDR of 0.9%). In our experiment, more genes (475) were downregulated than were upregulated (51) in the *ace2*ΔΔ mutant; again, this was similar to that found by Mulhern et al. (16) as were the gene ontology (GO) terms and gene groups affected.

We also isolated RNA from *cbk1*ΔΔ cells grown under the same conditions. Somewhat surprisingly, 1,006 genes were differentially expressed in the *cbk1*ΔΔ mutant relative to that in the WT during logarithmic yeast-phase growth, with 657 genes downregulated by at least 2-fold and 349 genes upregulated to the same extent (Fig. 1B; see also Table S4). These data indicate that Cbk1 has a significant effect on gene expression in C. albicans. As shown in the Venn diagram in Fig. 1C, only 70 genes are downregulated in both *ace2*ΔΔ and *cbk1*ΔΔ mutants (9.5% of Cbk1 and 13% of Ace2 sets). A list of the specific genes in each category of the Venn diagrams is provided in Table S6. The overlap between the genes upregulated in *cbk1*ΔΔ and *ace2*ΔΔ strains is even less (8 genes). Thus, Cbk1 and Ace2 directly or indirectly affect the expression of a significant number of genes during yeast-phase growth, but only a small fraction of these genes appears to dependent on the function of both proteins.

Consistent with previous single-gene analyses of both Cbk1 and Ace2, cell septum-degrading enzyme-encoding genes *CHT3* and *SCW11* (Fig. 1A and B) are among genes downregulated in both mutants. Of the remaining genes, ribosome/RNA processing (FDR, 0.00%) was the only GO term that was enriched in the set of genes differentially regulated in both *cbk1*ΔΔ and *ace2*ΔΔ mutants. These data, therefore, suggest that Cbk1-Ace2 primarily functions to regulate septum degradation during yeast growth, while both Ace2 and Cbk1 have significant wide-ranging effects on gene transcription that are independent of one another.

### Ace2 directly or indirectly represses the expression of a large set of genes during hyphal morphogenesis in Spider medium.

We also characterized the effect of an *ace2*ΔΔ mutation on gene expression during hyphal morphogenesis. *cbk1*ΔΔ mutants are unable to form hyphae (14, 19, 20); thus, we did not perform expression analysis under hypha-inducing conditions. Strains lacking *ACE2* form hyphae in liquid culture in response to a variety of standard inducing conditions and agents. Of these, Spider medium (SM) at 37°C is the only inducing condition under which *ace2*ΔΔ mutants differ from WT (32); *ace2*ΔΔ mutants form hyphae modestly slower than the WT. We, therefore, used this inducing condition rather than the serum induction that was employed by Mulhern et al. (16). In addition, we harvested cells after 4 h of induction, a time point where >75% of the *ace2*ΔΔ mutants have formed hyphae and Ace2 protein levels have peaked by Western blot analysis (32).

**FIG 2:**
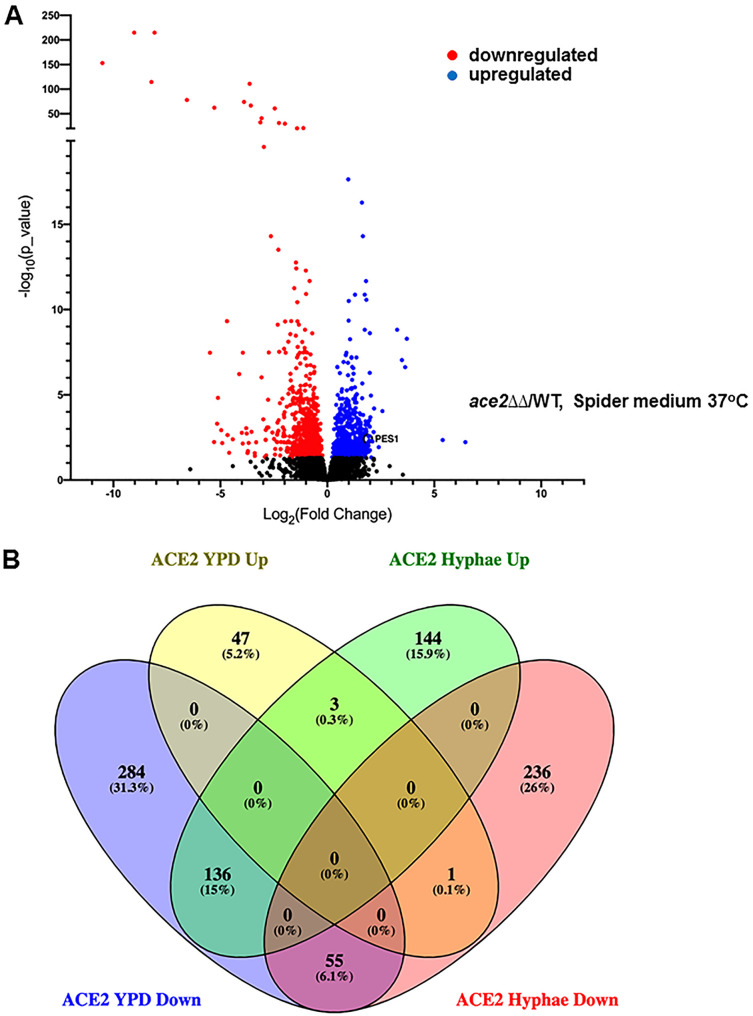
Ace2 directly or indirectly represses a large set of genes during hyphal morphogenesis. (A) Volcano plot of the gene expression profiles of *ace2*ΔΔ cells relative to that of the wild type after 4 h in Spider medium (SM) at 37°C, with genes showing statistically significant (adjusted *P* < 0.05) changes in expression of ±log_2_ of 1. (B) Venn diagram of differentially expressed genes (adjusted *P* < 0.05) for an *ace2*ΔΔ mutant relative to that of the wild type under yeast-phase and hyphal growth.

Under hypha-inducing conditions (SM, 37°C), a total of 575 genes were differentially expressed by at least 2-fold in the *ace2*ΔΔ mutant relative to that in the reference strain (Fig. 2A; see also Table S5). Interestingly, the set of genes was almost equally split between those with increased expression (283) and those with decreased expression (292). This is in distinct contrast to the relatively modest number of genes upregulated in *ace2*ΔΔ mutants during yeast-phase growth (Fig. 1A and C). This is also quite different from the number of upregulated genes (18 genes, 2-fold decreased) observed by Mulhern et al. (16) during hyphal induction with serum-containing medium. Nearly two-thirds of the genes upregulated in the *ace2*ΔΔ mutant are involved in the metabolism of carbon or nitrogen, while, as with during yeast-phase growth, approximately 40% of the genes are related to RNA/ribosome function.

It is important to note that very few genes are concordantly regulated by Ace2 during yeast and hyphal growth. Specifically, 54 genes were downregulated in both YPD and SM, while only 3 genes were upregulated under both conditions. Although the canonical Ace2 targets *SCW11*, *CST1*, and *DSE3* are among the sets of genes downregulated under both conditions, the only GO terms that were significantly enriched among the concordantly regulated genes are for carbon utilization (*ICL1*, *MLS1*, *PCK1*, *PDK2*, *PYC2*, and *SFC1*; FDR, 0.00%) and acetate metabolism (*CTN1*, *ICL1*, *MLS1*, and *SFC1*; FDR, 0.00%). Indeed, 136 genes were downregulated in *ace2*ΔΔ mutants in YPD at 30°C but were upregulated during hyphal induction in SM at 37°C. Once again, this set of genes is dominated by RNA/ribosome processing and metabolic functions. The large number of discordantly regulated genes emphasizes the context-dependent effect that Ace2 appears to have on C. albicans gene expression. Overall, these data indicate that Ace2 directly or indirectly affects the expression of many genes during hyphal morphogenesis in SM and that it appears to play a previously unappreciated role in repressing the expression of many genes under hypha-inducing conditions.

### Mutation of the Cbk1 phosphorylation sites in Ace2 by CRISPR/Cas9-mediated genome editing.

**FIG 3:**
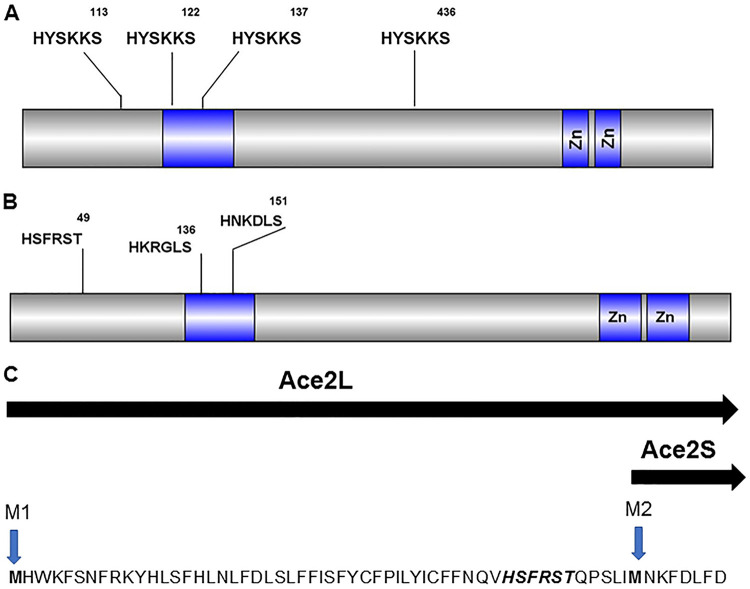
Schematic of putative Cbk1 phosphorylation sites and alternative isoforms of *Ca*Ace2. Schematic representation of Cbk1 phosphorylation motifs in S. cerevisiae (A) and C. albicans (B) Ace2. (C) Sequence of the N terminus of *Ca*Ace2 showing the two alternative AUG sites for initiation of translation and the resulting long (Ace2L) and short (Ace2S) forms as formulated by Calderón-Noreña et al. (23).

Previous phenotypic characterization of *ACE2* has shown that the C. albicans and S. cerevisiae orthologs have similar functions during yeast-phase growth and, in particular, are important for proper cell separation. As depicted in Fig. 3A, S. cerevisiae Ace2 (*Sc*Ace2) has four Cbk1 phosphorylation motifs, with two in the nuclear export sequence (NES), one N-terminal to that site, and one near the DNA-binding domain in the C-terminal region of the protein (10). The cell cycle functions of *Sc*Ace2 are dependent on S122, S137, and S436, indicating that *Sc*Cbk1 not only is required for concentration of *Sc*Ace2 in the nucleus through S122/S137 phosphorylation but also must contribute additional activation functions by phosphorylating S436 (10).

Ace2 (we use Ace2 to refer to the C. albicans ortholog and *Sc*Ace2 to refer to the S. cerevisiae ortholog for the remainder of the text) lacks the C-terminal Cbk1 phosphorylation motif but has sites in the NES and in the N-terminal region (Fig. 3B). Willger et al. performed a comprehensive phosphoproteomic study of C. albicans during hyphal induction and confirmed that Ace2 S136 and S151 are phosphorylated (30). There is evidence that in the SC5312 strain background (23), Ace2 has two isoforms, depending on the translational start site (Fig. 3B). The N-terminal Cbk1 phosphosite is between the two start sites and thus would only be expected to be present in the Ace2L form that is proposed to associate with the plasma membrane and play a role in septin dynamics. Ace2S is the isoform that localizes to the nucleus and regulates the expression of cell separation genes (23).

To assess the role of Cbk1 phosphorylation in Ace2 function, we used a CRISPR/Cas9 approach to mutate the serine and threonine Cbk1 consensus phosphorylation sites of the endogenous *ACE2* to alanines (33). The details of this strain construction are described in Materials and Methods. In this way, we constructed a strain in which the only *ACE2* allele lacks the Cbk1 phosphorylation sites in the NES (S136A and S151A; *ace2*-2A) and a strain that lacks all three of the Ace2 Cbk1 phosphorylation sites (S49A, S136A, and S151A; *ace2*-3A). The initially isolated phosphosite mutants were heterozygous at the *ACE2* locus, with a wild-type allele and the desired mutant allele. We deleted the wild-type allele using standard homologous recombination and confirmed that the only remaining allele contained the S/T-A mutations by Sanger sequencing. Thus, the resulting strains are heterozygous at the *ACE2* locus, and if the Cbk1 phosphorylation sites are required for function, the strains would be expected to have phenotypes that are similar to those from an *ace2*ΔΔ homozygous deletion. In our phenotypic characterization data presented below, the *ace2*ΔΔ mutant is used as the control. As shown in the supplemental material, *ace2*Δ/*ACE2* strains show no haploinsufficiency; thus, phenotypic changes are due to the mutations and not to changes in gene copy number (see Fig. S1).

10.1128/mBio.01900-20.1 FIG S1 Morphology of heterozygous *ace2*Δ/*ACE2* is not altered in comparison to that of the WT. (A) The indicated strains were spotted on a YPD plate, and the morphology was assayed after 3 days of incubation at 30°C. (B) *ace2*Δ/*ACE2* does not play a role in cell separation defect. The indicated strains were quantified for cell separation defect. Shown here are the percentages of groups of cells versus the indicated strains. Bars are the means ± SDs from three independent experiments; *n* ≥ 150 cells. (C) *ace2*Δ/*ACE2* is not hyperresistant to CFW. Serial dilutions of indicated strains were spotted on YPD medium and YPD with CFW (50 μg/ml), and the images were taken after 2 days of incubation at 30°C. (D) The heterozygote *ACE2* mutant is not critical for hyphal formation under embedded conditions. The indicated strains were embedded in YPS agar at 25°C, and images of colonies were taken after 2 days. Download FIG S1, TIF file, 0.3 MB. Copyright © 2020 Wakade et al. 2020 Wakade et al. This content is distributed under the terms of the Creative Commons Attribution 4.0 International license.

### Phosphoacceptor amino acids at Ace2 consensus Cbk1 substrate motifs are required for normal cell separation in C. albicans during yeast-phase growth.

**FIG 4:**
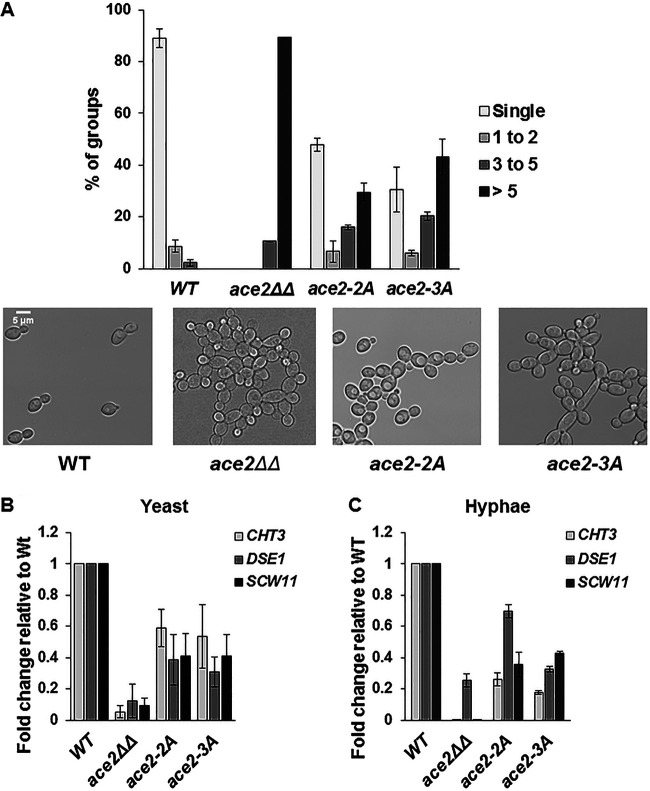
Cbk1 phosphoacceptor sites for cell separation and expression of septum-degrading enzymes. (A) Exponential-phase cultures of the indicated strains in YPD at 30°C were harvested, and the distribution of cell aggregates was determined by bright field microscopy, with the cells binned into the indicated groups. Representative photomicrographs of fields are shown for each strain. The bars indicate means from three independent experiments (*N* ≥ 150) with standard deviations (SDs) indicated by error bars. The differences in cell aggregates were statistically different from that for the wild type for all mutant strains (Student’s *t* test, *P* < 0.05) (B) The expression of the indicated Ace2 genes was determined by quantitative RT-PCR. The expression was normalized to *ACT1* by the 2^−ΔΔ^*^CT^* method and normalized to the WT. The bars indicate mean values for each strain, with error bars indicating the SDs from three independent experiments with technical replicates. The expression of each gene in the indicated mutants was statistically different from that in the WT (Student’s *t* test, *P* < 0.05).

A phenotype of *ACE2* and *CBK1* mutants that is conserved across all yeast species studied to date is decreased cell separation due to reduced septum degradation (8, 9, 15). Therefore, we compared the cell separation characteristics of the *ace2*-2A and *ace2*-3A mutants to those of the wild-type and *ace2*ΔΔ mutant strains under standard yeast culture conditions (30°C, YPD medium). The harvested cells were fixed and examined by light microscopy to determine the relative proportions of small, medium, and large cell clusters (Fig. 4A). Consistent with previous literature (8, 9, 15), WT cultures were dominated by small clusters (1 to 2 cells), while the *ace2*ΔΔ mutant cultures were almost entirely composed of large cell clusters (>5 cells) (Fig. 4A). The *ace2*-2A mutant showed an intermediate phenotype, with increased numbers of medium and large cell clusters that were not changed significantly by mutation of the non-NES Cbk1 site (*ace2*-3A). As previously found for *ace2*ΔΔ mutants (8, 9, 15), ultrasonication of the cell clusters formed by the *ace2*-2A and *ace2*-3A mutants did not alter the size of the clusters, which is consistent with a failure of the cells to separate and is inconsistent with noncovalent aggregation (data not shown).

As noted above, this phenotype is attributed to the decreased expression of septum-degrading enzymes such as *CHT3*, *DSE1*, and *SCW11* in the *ace2*ΔΔ mutant (Fig. 4B and C). The expression of these genes was also decreased in both *ace2*-2A and *ace2*-3A mutants during both yeast and hyphal cell growth (Fig. 4B and C). As with the cell separation data, mutations within the NES site are responsible for the majority of the observed effects on septum-degrading gene expression. This is consistent with the findings that the Ace2S form of the protein is responsible for transcriptional regulation of cell separation genes, while the site in the Ace2L form does not play a significant role in the expression of these genes (23). These data are consistent with previous studies in S. cerevisiae indicating that Cbk1 phosphorylation of the NES in Ace2 is required for expression of cell septum-degrading enzymes and proper cell separation (10).

### Cbk1 phosphoacceptor sites in a putative nuclear export signal site are required to concentrate Ace2 to daughter cell nuclei in C. albicans.

In S. cerevisiae daughter cells, phosphorylation of *Sc*Cbk1 consensus motifs in the NES of *Sc*Ace2 prevents its export and thereby concentrates *Sc*Ace2 within the nuclei of new daughter cells, while it is relatively excluded from mother cell nuclei (10). Mutations that prevent *Sc*Cbk1-mediated phosphorylation of *Sc*Ace2 result in the transcription factor localizing to both daughter and mother cell nuclei (10). To determine if a similar process occurs in C. albicans, we tagged the C terminus of *ACE2* in wild-type and *ace2*-*2A* strains with green fluorescent protein (GFP); we and others have shown previously that WT alleles retain function (15, 34), and we confirmed that the phenotypes of the *ace2*-2A-GFP strains did not differ from those of the parental strain (see Fig. S2).

10.1128/mBio.01900-20.2 FIG S2 Functionality of WT-Ace2-GFP and *ace2*-2A-GFP is not altered in comparison to that of the WT or untagged *ace2-2A* strain. (A) The indicated strains were spotted on YPD plates, and the morphology was assayed after 3 days of incubation at 30°C. (B) The indicated strains were quantified for the cell separation defect. Shown here are the percentages of groups of cells versus the indicated strains. Bars are the means ± SDs from three independent experiments. *n* ≥ 150 cells. Download FIG S2, TIF file, 0.1 MB. Copyright © 2020 Wakade et al. 2020 Wakade et al. This content is distributed under the terms of the Creative Commons Attribution 4.0 International license.

**FIG 5:**
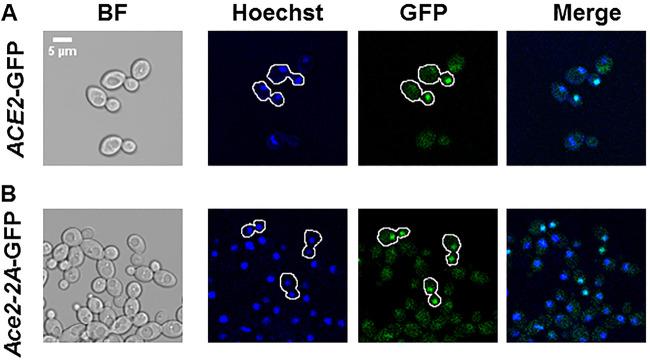
Cbk1 phosphoacceptor sites in the putative nuclear export site of Ace2 are required for exclusive localization to daughter cell nuclei in yeast-phase cells. Exponential cells for the *ACE2*-GFP (A) and *ace2-*2A-GFP (B) were harvested and stained with Hoechst before imaging in the indicated channels. The outlines of the indicated cells were generated to highlight mother-daughter pairs.

Consistent with previous reports, WT Ace2 localized to the daughter cell in budding yeast phase (Fig. 5A). In contrast, *ace2*-2A-GFP signal was present in both cells of mother-daughter pairs (Fig. 5B). This same pattern of mislocalization is consistent with that reported by the Weiss lab for strains containing *ScACE2* alleles lacking Cbk1 phosphoacceptor sites in the NES domain of the protein (10). This localization pattern also provides an explanation for the intermediate cell separation phenotype (relative to the *ace2*ΔΔ strain) observed for the *ace2*-2A strains, because some cells localized Ace2 to the daughter cell nuclei, whereas in the null mutant, there is a complete absence of protein. We attempted to characterize the effect of the *ace2*-2A allele on localization in hyphae, but the relatively few wild-type hyphae that showed the canonical Ace2 localization to the distal most nuclei of the hyphae prevented our ability to make firm conclusions (R. S. Wakade and D. J. Krysan, unpublished results). These data indicate that, as in S. cerevisiae, Cbk1 phosphorylation of Ace2 within the putative NES is required to concentrate Ace2 in daughter cell nuclei during yeast-phase growth.

### Cbk1 phosphorylation of Ace2 affects susceptibility to chitin-targeted cell wall stressors.

**FIG 6:**
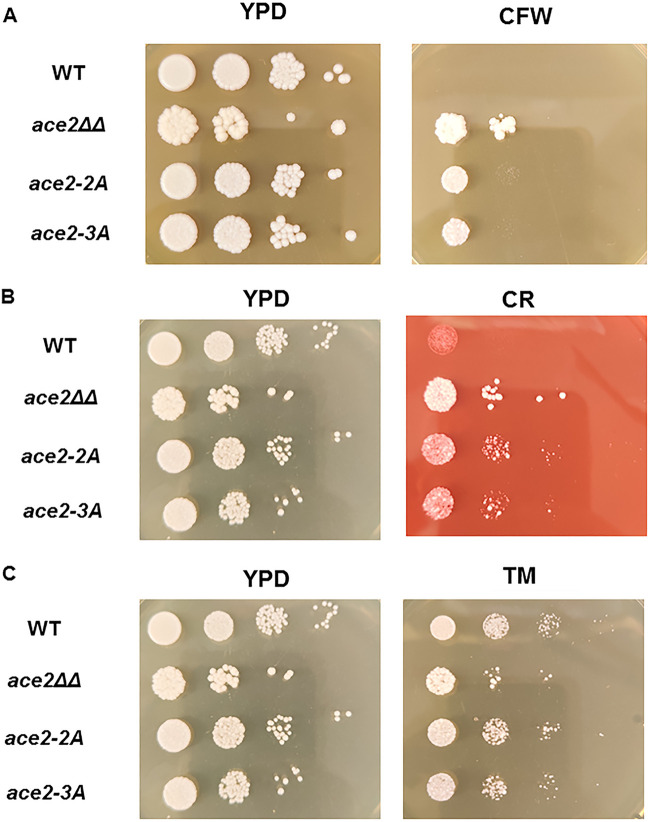
Cbk1 phosphoacceptor site mutants of *ACE2* are resistant to chitin-binding molecules but not tunicamycin. A 10-fold dilution series (OD_600_ of 0.1 for initial cell density) was plated on YPD as well as YPD containing calcofluor white (50 μg/ml) (A), Congo red (400 μg/ml) (B) and tunicamycin (4 μg/ml) (C). The plates were incubated for 3 days at 30°C before being photographed. The images are representative of two to three independent replicates.

Ace2 function has been implicated in maintaining cell wall homeostasis through multiple pathways (7). First, *ace2*ΔΔ mutants are resistant to the chitin-binding molecule calcofluor white (CFW). This is most likely due to an increase in the chitin content of the wall in the region of the septum due to decreased expression of chitinases. Consistent with our observation that the *ace2*-2A/3A mutants have decreased expression of *CHT3*, they are also resistant to concentrations of CFW that inhibit the growth of WT cells (Fig. 6A); similarly, both *ace2*ΔΔ and the *ace2*-2A/3A mutants are resistant to Congo red, a molecule that interacts with both chitin and glucan components of the cell wall (Fig. 6B). Ace2 has also been shown to affect the mannoprotein layer of the cell wall through a separate signaling pathway involving Cek1 (21); a manifestation of this function is the increased susceptibility of the *ace2*ΔΔ mutant to the glycosyl-transfer inhibitor tunicamycin (Fig. 6C). The *ace2*-2A/3A mutants grew similarly to wild-type cells at concentrations of tunicamycin that inhibits *ace2*ΔΔ growth, indicating that this function of Ace2 is independent of Cbk1. Thus, the cell wall functions regulated by Cbk1-Ace2 appear to be mainly limited to cell septum-related processes, while other cell wall functions of Ace2 are independent of Cbk1.

### Cbk1 phosphoacceptor mutants have context-specific effects on the role of Ace2 under filament-inducing conditions.

**FIG 7:**
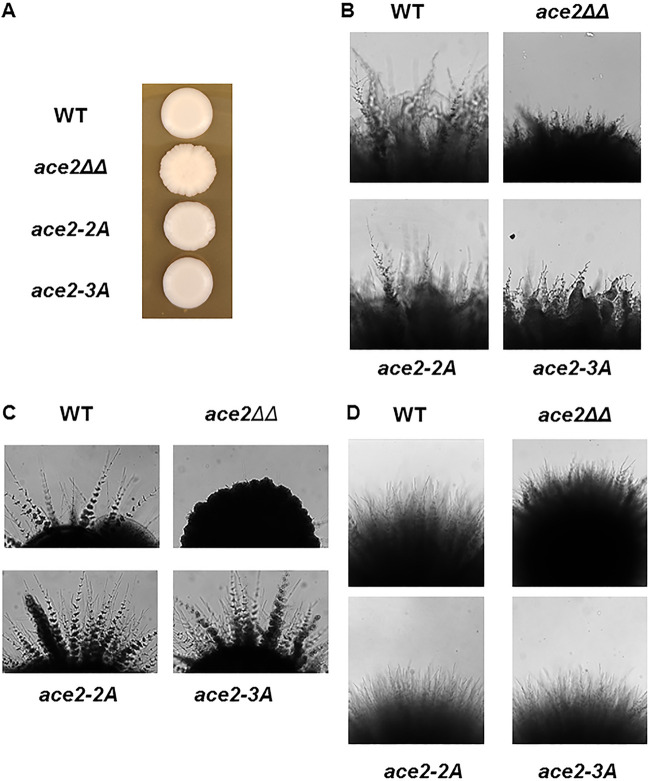
Cbk1 phosphorylation of Ace2 has a modest effect on hyphal formation under hypoxic conditions. (A) The scalloped colony morphology of the *ace2*ΔΔ mutant is not phenocopied by the Cbk1 phosphoacceptor site mutants *ace2-*2A and *ace2*-3A after 3 days at 30°C on YPD. (B) Cbk1 phosphoacceptor site mutants *ace2*-2A and *ace2*-3A have modestly reduced hyphal formation under hypoxic conditions (0.1% O_2_; surface inoculation, YPS; 2 days). The Cbk1 phosphorylation sites of Ace2 are not required for hyphal morphogenesis under embedded conditions (YPS) at 25°C (C) or 37°C (D), but *ACE2* is required for hyphal formation at 25°C.

The role of Ace2 during C. albicans hyphal morphogenesis is dependent on the environmental conditions that induce the morphological transition. Under noninducing conditions, cultures of *ace2*ΔΔ mutants have increased numbers of pseudohyphal cells (15, 16). As a result, the colony morphology of *ace2*ΔΔ mutants on YPD plates at 30°C takes on a wrinkled or scalloped appearance, while wild-type strains form typical smooth colonies (Fig. 7A). This well-described phenotype suggests that Ace2 may play a role in suppressing the hyphal morphogenesis program in daughter yeast cells under noninducing conditions. As shown in Fig. 7A, the colony morphology of the *ace2*-2A/*ace2*-3A mutant is similar to the smooth surface of WT strains, indicating that Cbk1 plays a minor role in this function of Ace2.

Both the Butler and Ernst groups have shown that Ace2 is required for hyphal formation under hypoxic conditions (16, 17). As shown in Fig. 7B, in the SN strain background, Ace2 is not absolutely required for hyphal formation under hypoxic conditions, but the *ace2*ΔΔ mutant strain clearly is deficient in hyphal formation relative to the reference strain. The *ace2*-2A and *ace2*-3A mutants also undergo less robust hyphal morphogenesis relative to that of the reference strain under hypoxic conditions, suggesting that the Cbk1-Ace2 pair plays a modest role during hypoxia-induced hyphal formation. Mulhern et al. (16) also reported that *ace2*ΔΔ mutants are dramatically deficient for hyphal formation when embedded in yeast-peptone-sucrose (YPS) at 25°C, and we observed the same phenotype for our *ace2*ΔΔ strains (Fig. 7C). Interestingly, the *ace2*-2A and *ace2*-3A mutants formed hyphae robustly under these conditions (Fig. 7C). Consistent with the data of Mulhern et al. (16), WT as well as the *ace2*-2A/*ace2*-3A mutants formed hyphae when embedded in YPS and incubated at 37°C instead of 25°C (Fig. 7D). These data indicate that the role of Ace2 in supporting the formation of hyphae is largely independent of Cbk1.

### Identification of a novel Ace2 function during hyphal morphogenesis: Cbk1-dependent suppression of the hypha-to-yeast transition.

**FIG 8:**
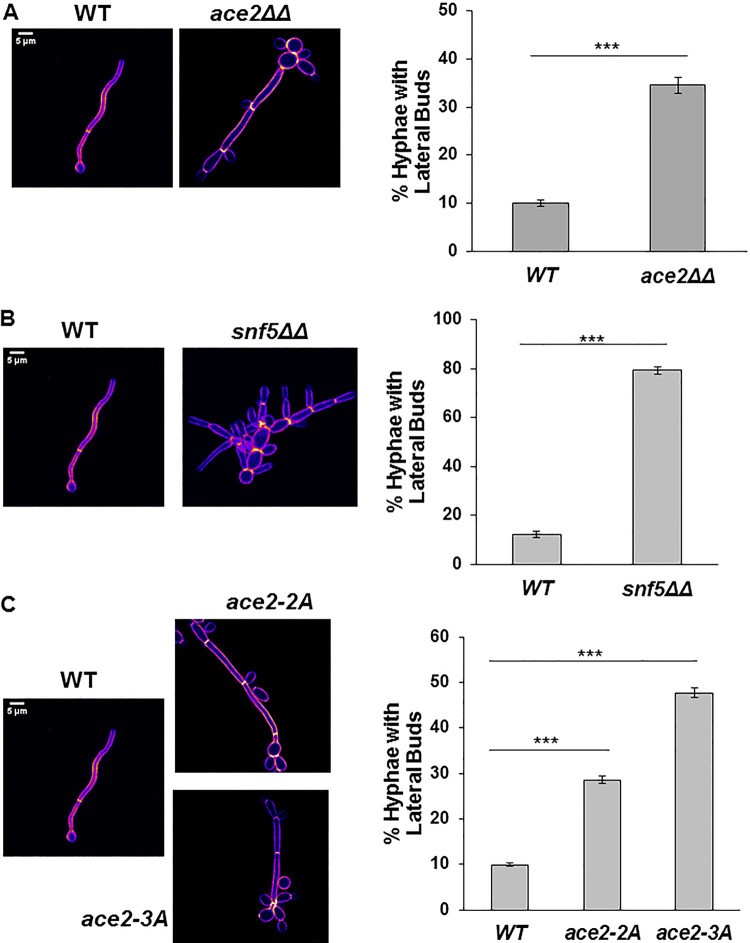
Ace2 and Snf5 suppress the hypha-to-yeast transition in a Cbk1-dependent manner. The indicated strains were induced to form hyphae in Spider medium (SM) at 37°C for 4 h. The cells were fixed, and the percentage of hyphae with yeast buds at subapical compartments was determined. The bars indicate the means from three independent experiments (*N* ≥ 100), with SDs indicated by the error bars. Representative images of calcofluor white-stained cells of WT and *ace2*ΔΔ (A), *snf5*ΔΔ (B), *ace2-*2A/3A (C) mutant are shown.

We previously reported that *ace2*ΔΔ mutants undergo hyphal morphogenesis in liquid media containing a variety of inducing agents (32), although the tempo of hyphal formation is modestly slower in SM. Upon reexamination of this process, we noted that *ace2*ΔΔ strains developed lateral yeast cells at subapical segments of the hyphal filament before they were evident on WT hyphae (Fig. 8A). In addition, we observed yeast phase budding from the mother cells from which the hyphal structure emerged, indicating that in the absence of *ACE2*, hyphal mother cells had reentered the cell cycle earlier than WT cells (Fig. 8A). As hyphae mature, a hypha-to-yeast transition eventually occurs, leading to the emergence of yeast buds at subapical cell compartments within the hyphae (31, 35). Quantification of this observation confirmed that at 4 h of induction, only 10% of WT hyphae displayed lateral yeast cells at subapical segments, whereas 35% of *ace2*ΔΔ mutants had formed lateral yeast cells (Fig. 8B). Finkel et al. had previously reported that Snf5 regulates *ACE2* expression in SM, and our inspection of photomicrographs of *snf5*ΔΔ mutants suggested that there may be increased lateral yeast formation in these strains as well (18). Consistent with that assessment, 80% of hyphae formed by the *snf5*ΔΔ mutant had lateral yeast at a time point when wild-type cells had only 10% (Fig. 8B). Our data indicate that Ace2 and Snf5 repress lateral yeast formation and further suggest that Snf5 and Ace2 are required to ensure that the transition from hyphae to yeast does not occur prematurely. In addition, it appears that *ace2*ΔΔ is required to prevent hyphal mother cells from reentering the cell cycle prematurely.

We next asked whether Cbk1 was required for the ability of Ace2 to suppress lateral yeast cell formation during the maintenance phase of hyphal formation. As shown in Fig. 8C, the *ace2*-2A and *ace2*-3A cells showed increased lateral bud cell formation, indicating that this Ace2 function is likely to be Cbk1 dependent. The extent of lateral yeast cell formation is also increased in the *ace2*-3A mutant relative to that in the *ace2*-2A mutant (Fig. 8C). Phosphorylation at S49 is only relevant if the Ace2L isoform plays a role (Fig. 3B); if Ace2S was the operative isoform, then the phenotypes of *ace2*-2A and *ace2*-3A should be identical (23). Taken together, these data indicate that the Cbk1-Ace2 axis functions to suppress the hypha-yeast transition during the maintenance phase of hyphal morphogenesis in C. albicans.

### Expression of *PES1*, a regulator of the hypha-to-yeast transition, is increased in *ace2*ΔΔ and *ace2* phosphosite mutants.

**FIG 9:**
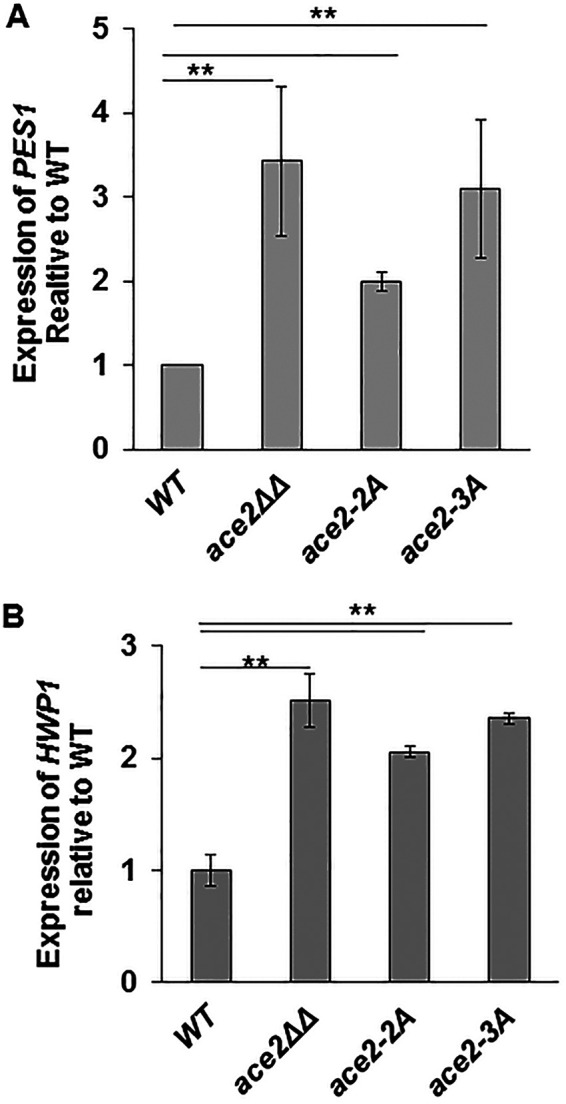
The expression of markers of *PES1* and *HWP1* is increased in *ace2*ΔΔ and *ace2-*2A/3A mutants. The indicated strains were induced to form hyphae in Spider medium (SM) at 37°C for 4 h and harvested, and RNA was isolated. The expression of *PES1* (A) and *HWP1* (B) was determined by quantitative RT-PCR and normalized to *ACT1* in each mutant. The relative expression of each gene was further normalized to that of the WT. The bars indicate mean values for each strain, with error bars indicating the SD, from three independent experiments with technical replicates. The expression of each gene was statistically different from WT (Student’s *t* test, *P* < 0.05).

The hypha-to-yeast transition has not been studied to nearly the same extent as the yeast-to-hypha transition (31, 35). In pioneering work, the Köhler lab found that *PES1*, a pescadillo homolog, is required for the hypha-to-yeast transition and is essential in yeast-phase cells but not during hyphal phase growth (31). Conversely, increased expression of *PES1* induces increased lateral yeast formation. We therefore examined our RNA sequencing data set to determine the expression level of *PES1* in *ace2*ΔΔ mutants. Indeed, *PES1* expression was increased 3.8-fold in hyphal *ace2*ΔΔ mutants relative to that in the wild type but was actually reduced in yeast *ace2*ΔΔ cells (Tables S3 and S5); the same trend was observed by Mulhern et al. in their transcriptional profile of *ace2*ΔΔ mutants (16). To confirm this observation, the expression of *PES1* was measured by reverse transcription-quantitative PCR (qRT-PCR) in WT and *ace2*ΔΔ strains after 3 h of induction with SM (Fig. 9A). The expression of *PES1* was increased in the *ace2*ΔΔ strain relative to that in the wild type, confirming the genome-wide expression profiling data and strains containing Ace2 Cbk1-phosphosite mutations also have increased expression of *PES1* (Fig. 9A). Consistent with the increased lateral yeast formation in the *ace2*-3A strain relative to that in *ace2-*2A mutants, the *ace2*-3A mutant showed a trend toward increased *PES1* expression relative to that in the *ace2-*2A mutant (Fig. 8B). Interestingly, the archetypal hyphal specific gene *HWP1* was expressed at increased levels in the *ace2*ΔΔ deletion and the Cbk1 phosphosite mutants as well (Fig. 9C), indicating that despite increased development of lateral yeast, the hyphal transcriptional program is still strongly induced. Thus, the expression of well-characterized reporter genes for both the yeast-to-hypha and the hypha-to-yeast transcriptional programs is upregulated in *ace2* mutants. This expression profile suggests that the Cbk1-Ace2 axis plays an important role in regulating the balance between morphogenic transcriptional programs during hyphal formation in C. albicans.

## DISCUSSION

The RAM pathway regulates a wide range of processes in C. albicans, including cell cycle-associated daughter cell separation, hyphal morphogenesis, cell wall integrity and biosynthesis, biofilm formation, oxidative stress resistance, and mammalian infection (14–20). Although the phosphorylation motif of the key kinase in the RAM pathway, Cbk1, has been well described (10), the substrates that carry out the effector functions of the pathway remain largely uncharacterized in C. albicans with the exceptions of Bcr1, Ssd1, and Fkh2 (27–29). For example, prior to the work described above, it was unclear what functions of the RAM pathway were due to its eponymous transcription factor Ace2.

Through transcriptional profiling of both *cbk1*ΔΔ and *ace2*ΔΔ mutants and genetic analysis of strains containing mutations at the Cbk1 phosphosites, we have found that, although both Cbk1 and Ace2 affect the expression of a large number of genes and have a number of phenotypes, the Cbk1-Ace2 axis directly regulates a specific subset of these functions. During yeast-phase growth, the expression of only ∼10% genes was concordantly affected in *cbk1*ΔΔ and *ace2*ΔΔ mutants. The cell septum-degrading enzymes encoded by *CHT3* and *SCW11*, well-characterized effectors of the RAM pathway (7), are among these and are also downregulated in Cbk1-phosphosite mutants of *ACE2*. As expected from studies of Cbk1 and Ace2 in S. cerevisiae, the *ACE2* phosphosite mutants have partial cell separation defects and alterations in the daughter cell-specific nuclear localization of Ace2 (10).

The small overlap between the expression profiles of *cbk1*ΔΔ and *ace2*ΔΔ mutants indicates that Cbk1 is likely to have additional transcriptional effectors and that Ace2 is likely to have other regulatory partners. With regard to the former, Bcr1 (27) and Fkh2 (28) have been shown to be Cbk1-regulated transcription factors. Bcr1 is important for biofilm formation, but its effect on gene expression during yeast phase growth has not, to our knowledge, been characterized (36). Fkh2, interestingly, plays a role in both hyphal morphology and cell separation. During yeast-phase growth, the expression of *SCW11* and *CHT2* is reduced in *fkh2* mutants (28). It is therefore possible that Cbk1 regulates cell separation genes through both Fkh2 and Ace2 and that other Cbk1-regulated genes are Fkh2 targets. A survey of the annotated C. albicans transcription factors indicates that an additional 27 have sequences that match Cbk1 phosphorylation motifs. Of these, a highly likely Cbk1 substrate is Ash1. Like Ace2, it is also a daughter cell-associated transcription factor that plays a role in hyphal formation under specific conditions. Its expression is also dependent upon both Cbk1 and Ace2 (Tables S2 and S3). Ash1 has an HTRSRS Cbk1-consensus sequence in the N terminus, and S22 is phosphorylated during both yeast- and hyphal-phase growth according to two large-scale phosphoproteomic studies in C. albicans (30, 37).

It is also likely that another contributing factor to the role of Cbk1 in gene expression is its regulation of Ssd1, an RNA-binding protein that suppresses the expression of a specific set of genes (29). In S. cerevisiae, *Sc*Cbk1 phosphorylates *Sc*Ssd1 to suppress its ability to target bound mRNAs to the P-body for storage and/or degradation (12, 38, 39). This set of S. cerevisiae genes is enriched in cell wall and cell separation genes but contains others as well (11, 38). In C. albicans, the only transcript that has been shown to be affected by Cbk1-Ssd1 is Nrg1, which is downregulated at the initiation of hypha formation (29). It is unlikely that this is the only gene that is targeted by Ssd1, although additional studies will be needed to identify these transcripts. Overall, Cbk1 has a broad effect on C. albicans gene expression through a combination of its modulation of Ace2 activity, regulation of other transcription factors, and inhibition of Ssd1.

Ace2 also has broad effects on gene expression beyond cell separation as noted above (Fig. 1 and 2; see also Tables S1 and S3 in the supplemental material). Willger et al. found that Ace2 is phosphorylated at 16 sites in addition to those that match the Cbk1 motif, suggesting that other kinases are likely to be involved in the regulation of Ace2 function (30). Indeed, genetic studies by van Wijlick et al. indicate that Ace2 functions in a kinase cascade composed of Cst20, Hst7, and Cek1 to regulate protein mannosylation and susceptibility to the glycosylation inhibitor tunicamycin (21). Our data are consistent with their model in that the hypersusceptibility of the *ace2*ΔΔ mutant to tunicamycin is not recapitulated by *ACE2* mutants lacking Cbk1 phosphorylation sites (Fig. 6).

10.1128/mBio.01900-20.3 TABLE S1 Strains used in this work. Download Table S1, DOCX file, 0.1 MB. Copyright © 2020 Wakade et al. 2020 Wakade et al. This content is distributed under the terms of the Creative Commons Attribution 4.0 International license.

10.1128/mBio.01900-20.4 TABLE S2 Oligonucleotides used in this work. Download Table S2, XLSX file, 0.1 MB. Copyright © 2020 Wakade et al. 2020 Wakade et al. This content is distributed under the terms of the Creative Commons Attribution 4.0 International license.

10.1128/mBio.01900-20.5 TABLE S3 RNA sequencing data for WT and *ace2*ΔΔ strains in YPD at 30°C. Download Table S3, XLSX file, 0.3 MB. Copyright © 2020 Wakade et al. 2020 Wakade et al. This content is distributed under the terms of the Creative Commons Attribution 4.0 International license.

10.1128/mBio.01900-20.6 TABLE S4 RNA sequencing data for WT and *cbk1*ΔΔ strains in YPD at 30°C. Download Table S4, XLSX file, 0.4 MB. Copyright © 2020 Wakade et al. 2020 Wakade et al. This content is distributed under the terms of the Creative Commons Attribution 4.0 International license.

10.1128/mBio.01900-20.7 TABLE S5 RNA sequencing data for WT and *ace2*ΔΔ strain in Spider medium at 37°C. Download Table S5, XLSX file, 0.3 MB. Copyright © 2020 Wakade et al. 2020 Wakade et al. This content is distributed under the terms of the Creative Commons Attribution 4.0 International license.

10.1128/mBio.01900-20.8 TABLE S6 List of genes for each Venn diagram category. Download Table S6, XLSX file, 2.0 MB. Copyright © 2020 Wakade et al. 2020 Wakade et al. This content is distributed under the terms of the Creative Commons Attribution 4.0 International license.

Desai et al. have also performed chromatin immunoprecipitation sequencing (ChIP-seq) with Ace2 in YPD at 30°C under normoxia and hypoxia (17); because these data were not correlated with expression profiling, we compared the set of genes downregulated in the *ace2*ΔΔ mutant to the set of genes Desai et al. found to be bound by Ace2-hemagglutinin (HA) under the same conditions. Surprisingly, only 23 of 359 (6.4%) genes identified by Ace2-HA ChIP were downregulated in the *ace2*ΔΔ mutant under these conditions. Further inspection of the ChIP data set revealed that *SCW11*, *CHT3*, and *DSE3*, genes previously confirmed by single-gene ChIP (40), were not among those identified as interacting with Ace2 during yeast-phase growth. The reasons for the lack of overlap between these two large data sets are not clear.

Promoter motif analysis reported by Desai et al. (17) identified a motif that was previously deduced by Mulhern et al. (16); however, only 11 of the genes reported by Mulhern et al. to be downregulated in the *ace2*ΔΔ mutant were bound by Ace2-HA (17). One possibility is that the HA tag is altering the function of Ace2 in a manner that is not evident from phenotypic analysis; although, previous studies have shown that C-terminally tagged Ace2 proteins bind to targets such as *SCW11* and *CHT3* (40). It seems from these data and observations that additional experiments are needed before a definitive conclusion can be made regarding direct targets of Ace2 and the binding motifs that determine such targets.

Under many conditions, Ace2 is dispensable for C. albicans hyphal morphogenesis. Despite this, transcriptional profiling data reported by Mulhern et al. (16) and reported herein clearly indicate that it affects the expression of many genes during hyphal morphogenesis (Fig. 2; Table S3). Our data indicate that a significant set of genes are upregulated in the absence of Ace2, suggesting that it functions either directly or indirectly as a repressor of gene expression during hyphal growth. Interestingly, *HWP1*, a gene that is only expressed during hyphal growth, and *PES1*, a gene associated with hypha-to-yeast transition, are both upregulated (Fig. 9A and B). *PES1* is essential during yeast-phase growth and promotes the hypha-to-yeast or lateral bud formation in hyphal cells (31); it is not essential for hyphal growth, and forced expression drives lateral yeast cell formation in hyphae. Our data indicate that loss of Ace2 function and loss of Cbk1 regulation of Ace2 lead to increased expression of *PES1* and early formation of lateral yeast bud or hypha-to-yeast transition (Fig. 8 and 9). To our knowledge, this represents a novel function of Ace2 during morphogenesis, and as such, Ace2 is one of only a small set of genes that have been demonstrated to affect the hypha-to-yeast transition (31, 38, 39).

We propose that the Cbk1-Ace2 axis functions during the maintenance phase of hyphal formation to suppress the lateral yeast growth program. During hyphal morphogenesis, Ace2 RNA and protein levels are initially low and then increase to peak at approximately 5 h postinduction in SM (32). Thus, except under specific conditions, Cbk1-regulated Ace2 does not appear to be required for initiation of hyphal formation. However, Ace2 appears to play an important role in subapical compartments by maintaining the hyphal transcriptional program or, alternatively, suppressing the yeast program (see below).

We and others have shown that Ace2-GFP is most clearly localized to the nuclei of the leading hyphal tip (15, 34). In addition to this well-established role in daughter cells, our data indicate that Cbk1-regulated Ace2 also functions in subapical hyphal cells as well. Although we have not been able to observe Ace2-GFP signal in the nuclei or cytoplasm of subapical hyphal cells (R. S. Wakade and D. J. Krysan, unpublished data), we suspect that this is due to the low overall expression of Ace2 from its native promoter; indeed, long exposure times are needed to visualize Ace2-GFP in the nuclei of daughter yeast cells or hyphal tip cells in our experience. The phenotypic data for the *ace2*ΔΔ and *ace2-*2A/3A strains, however, provide compelling evidence that Ace2 functions in the subapical and mother cell compartments of the hyphae.

Interestingly, our genetic data also indicate that the recently described Ace2L isoform may be required for this function (Fig. 3, 8, and 9) (23). Because Calderón-Noreña et al. found that the Ace2S isoform is likely responsible for much of the transcriptional regulation attributed to Ace2 (23), it is possible that suppression of the yeast program is not a direct result of Ace2 binding to DNA; indeed, Ace2L is proposed to be membrane associated. Clearly, additional work will be needed to fully characterize the mechanistic details of this newly described Ace2 function.

Regardless of the specific molecular mechanism, the function of Ace2 in the subapical and mother cell compartments of the hyphal structure is consistent with its known cell cycle functions during yeast-phase growth (9, 10) and the proposed cell cycle state of subapical compartments of C. albicans hyphae (6, 41). Specifically, Ace2 is well established to play a critical role in early G_1_ during yeast-phase growth in both S. cerevisiae (9, 10, 42) and C. albicans (43). As the cell progresses through G_1_, the amount of nuclear Ace2 is reduced by decreased *Sc*Cbk1 phosphorylation (42) and *Sc*Amn1-mediated ubiquitin-mediated degradation of Ace2. This decrease in *Sc*Ace2 is associated with the transition to yeast cell cycle checkpoint (Start) (44). In contrast to that during yeast growth, Ace2 protein levels increase as hyphal morphogenesis progresses (32), suggesting that hyphal cells maintain an early G_1_-like state. Interestingly, *CaAMN1* expression is downregulated >3-fold relative to that in yeast cells after 4 h of SM induction (Tables S1 and S3), suggesting that decreased Amn1-mediated proteasome degradation of Ace2 could contribute to the steady increase in its protein level as hyphal morphogenesis progresses (32).

Subapical and mother cells of hyphae have been shown to be arrested in G_1_ phase (6, 42). Gow and colleagues have developed a model in which the highly vacuolated nature of the subapical compartments limits the amount of cytoplasm, leading to an effectively smaller cell size (41, 45). Since cell size is a critical determinant of the cell transitioning from G_1_ to Start, lateral yeast cell or branching does not occur until the ratio of cytoplasm to vacuole increases. Our data indicate that Cbk1-phosphorylated Ace2 plays a role in maintaining G_1_ and inhibiting the transition to Start in both mother cells and subapical compartments. To our knowledge, this is the first genetic evidence for proteins that function to repress the transition from G_1_ to Start in C. albicans hyphae.

**FIG 10:**
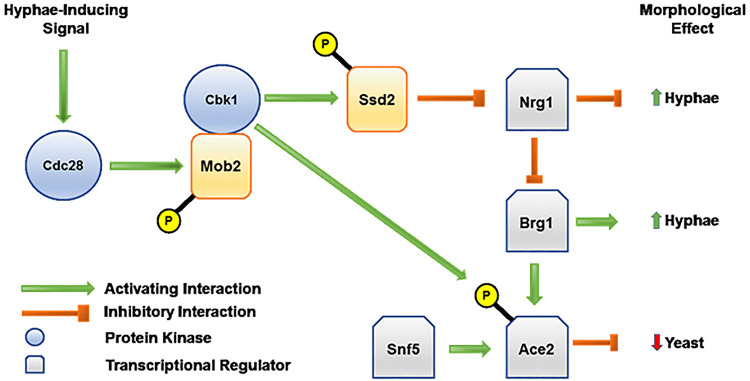
Model describing the function of the Cbk1-Ace2 axis during hyphal morphogenesis.

Taking these new observations together with previous work from multiple labs, including our own, allows us to construct the following model integrating the function of the Cbk1-Ace2 axis during hyphal morphogenesis under standard liquid medium induction conditions (Fig. 10). After a hypha-inducing signal is sensed, Cdc28 is activated (45) and, as demonstrated by Gutiérrez-Escribano et al. (46), phosphorylates the critical activator of Cbk1, Mob2. Based on work by Lee et al., Cbk1 phosphorylates Ssd1, which in turn leads to degradation of the mRNA for the repressor of hyphal morphogenesis, Nrg1 (29); decreased Nrg1 activates hyphal morphogenesis through a number of pathways (47). Of the proteins derepressed by decreased Nrg1 activity, the transcription factor Brg1 is particularly important (48). Indeed, Cleary et al. have shown that Nrg1 and Brg1 are part of a feedback loop that leads to increased *BRG1* expression during the initiation of hyphal morphogenesis (49).

We previously showed that Brg1 directly activates *ACE2* expression during hyphal morphogenesis and that expression peaks during the maintenance phase of the process (32). Finkel et al. found that Snf5 is also required for *ACE2* expression (18), and we show that it is also required for suppression of lateral yeast formation, indicating it plays a role in maintaining proper levels of *ACE2* during morphogenesis. Finally, as proposed above, Cbk1-phosphorylated Ace2 functions, at least in part, to suppress lateral yeast formation, likely through delaying the transition from G_1_ to Start in subapical hyphal cell compartments.

In summary, Cbk1 regulates a specific set of Ace2 functions during both yeast and hyphal-phase growth in C. albicans. While both Cbk1 and Ace2 have pleotropic effects on cell physiology, the majority of those effects are independent of one another. We have also proposed an integrated pathway through which Cbk1 affects hyphal formation under standard laboratory induction conditions, which involves its regulation of the transcriptional regulators Nrg1, Brg1, and Ace2. Lastly, our data indicate that Cbk1-Ace2 is required to delay the G_1_-Start transition in hyphae and, thereby, suppress the hypha-to-yeast transition.

## MATERIALS AND METHODS

### Cultivation conditions, media, and materials.

All Candida albicans strains were precultured overnight in yeast-peptone-dextrose (YPD) medium at 30°C, unless indicated otherwise. Standard recipes (48) were used to prepare synthetic drop-out media, YPD, Spider medium (SM), and yeast-peptone-sucrose (YPS). Strains were induced for hyphal morphogenesis in liquid medium by diluting an overnight culture into SM and shifting to 37°C (32). Hyphal induction on solid surfaces was carried out either on SM (50, 51) or embedded in top agar-YPS medium according to published protocols (16). Growth on YPD plates containing the indicated concentrations of calcofluor white, Congo red, and tunicamycin was examined as described (21). The chemical reagents were obtained from Millipore-Sigma and used as received.

### Strain construction.

Strains and oligonucleotides used in this study are listed in the Tables S1 and S2, respectively, in the supplemental material. All heterozygous or homozygous strains of C. albicans were constructed from an SN152 background using the auxotrophic marker *LEU2* or *ARG4.* The *ace2*Δ*/ACE2* strain was generated as described previously (26). Briefly, pCR4-TOPO plasmid carrying the *ACE2*::*LEU2* amplicon was digested with the SbfI enzyme, and subsequently, the linearized plasmid was further inserted into the SN87 background (52). The transformants were selected on SD plates lacking leucine, and correct integration was confirmed by PCR using ACE2.P1 and ACE2.P2 primers.

The transient CRISPR-Cas9 system was used to generate strains with deletion mutations (33). To generate *ace2*ΔΔ strains, a disruption cassette with 5′ and 3′ flanking sequences homologous to the 5′ and 3′ regions of *ACE2* was generated by amplification of *CmLEU2* cassette from the pSN40 plasmid (52) with primer pairs ACE2.P3 and ACE2.P4. The *CaCas9* expression cassette was PCR amplified from plasmid pV1093 (33) and using CAS9.P1 and CAS9.P2 primers, whereas a single guide RNA (sgRNA) expression cassette was generated using the split-joint PCR method (33). Briefly, in the first step, the SNR52 promoter was amplified using primer pairs ACE2.P5 and ACE2.P6, whereas the sgRNA scaffold was PCR amplified by using ACE2.P7 and ACE2.P8 primers. In the second step, the SNR52 promoter and sgRNA scaffold were fused by primer extension in which the 20-bp guide RNA sequences act as complementary primers. In the third round, the fused PCR product was PCR amplified by using the nested primers ACE2.P9 and ACE2.P10 to harvest the sgRNA cassette. For fungal transformation, 1 μg of *CaCas9* cassette and 1 μg sgRNA cassette were cotransformed with the 3 μg of the deletion construct by using the standard lithium acetate transformation method (33, 52).

To generate a strain with serine/threonine-to-alanine mutations at Cbk1 consensus phosphorylation sites, a fragment of *ACE2* (1 to 622 bp) that contained the respective mutations at T49A, S136A, and S151A was synthesized by GeneScript and cloned into pUC19. This fragment was used as a template to generate strains containing the *ace2*-2A (S136A and S151A) and the *ace2-*3A (T49A, S136A, and S151A) mutations. To do this, we used a double CRISPR approach. We first isolated the C. albicans *ARG4* gene from an SC5314 background, and PCR amplified it using ARG4.P1 and ARG4.P2 primers. An *ARG4* cassette targeted to the putatively neutral *dpl200* locus was amplified using ARG4.P3 and ARG4.P4 primers. The phosphodeficient *ACE2* allele was amplified by using ACE2.P11 and ACE2.P12 primers. To achieve the transformation, 1 μg of *CaCas9* cassette, 1 μg of each sgRNA-*ACE2* and sgRNA-*ARG4* cassette were cotransformed with the 3 μg of *ACE2*-2A/3A along with 1 μg of the *ARG4* cassette. The transformants were selected on synthetic media lacking Arg.

To characterize the resulting transformants, the region of *ACE2* corresponding to the repair fragment was PCR amplified using ACE2.P11 and ACE2.P12 primers and analyzed by Sanger sequencing. From multiple transformations, the majority of isolates were heterogeneous, comprising one copy of WT-ACE2 whereas other copy contained two (S136A and S151A) or three (T49A, S136A, and S151A) mutations in *ACE2*. We then deleted the remaining WT copy of *ACE2* as described earlier (26), and the transformants were selected on SD plates lacking leucine and arginine. The correct integration was confirmed by PCR using ACE2.P1 and ACE2.P2 primers, and the presence of the *ACE2* mutations was confirmed by Sanger sequencing. In this way, we generated strains in which the only copy of *ACE2* contained mutations in the Cbk1 phosphorylation sites.

Strains with GFP fused to the C terminus of *ACE2* were generated by homologous recombination using a cassette that was derived from pMG2120 (53) and primers ACE2.P13 and ACE2.P14. The PCR amplified *NAT1*-marked GFP cassette was purified and transformed into either the SN152 reference strain or *ACE2*-2A strain. Two independent clones of the respective strain were generated, and correct integration was confirmed by PCR. The resulting strains showed no change in growth or morphogenesis phenotypes relative to those of the parental strains. All PCR-amplified or cloned fragments were confirmed by sequencing. Correct insertions of two independent clones were verified by PCR and used for the further experiments. Oligonucleotides were synthesized by IDT Technologies (Coralville, IA) and used as received.

### RNA sequencing.

The indicated strains were grown overnight in YPD at 30°C, back diluted the next day, and grown to exponential phase (WT and *ace2*ΔΔ and *cbk1*ΔΔ mutants) or induced to form hyphae with SM (WT and *ace2*ΔΔ strains) at 37°C for 4 h. The cells were collected and centrifuged for 2 min at 11,000 rpm, and RNA was extracted from the pellet according to the manufacturer’s protocol (MasterPure Yeast RNA purification kit, catalog number MPY03100). Briefly, the pellet was resuspended in 300 μl of Extraction reagent containing proteinase K (50 μg/μl), and the mixture was incubated at 70°C for 15 min. The samples were placed on ice for 5 min, and the cell debris was precipitated by addition of 175 μl of MPC reagent and centrifuged for 10 min at 11,000 rpm at 4°C. The supernatant was transferred to the new tube, and 500 μl of ice-cold isopropanol was used for mRNA precipitation. The obtained pellet was washed twice with ice-cold 70% ethanol and dried, and mRNA was resuspended in 35 μl of Tris-EDTA (TE) buffer.

Total RNA submitted to the University of Wisconsin—Madison Biotechnology Center was verified for purity and integrity via the NanoDropOne spectrophotometer and Agilent 2100 Bioanalyzer, respectively. Samples that met the Illumina sample input guidelines were prepared according the TruSeq Stranded mRNA Sample Preparation Guide (rev. E) using the Illumina TruSeq Stranded mRNA Sample Preparation kit (Illumina Inc., San Diego, CA, USA). For each library preparation, mRNA was purified from 1 μg total RNA using poly(T) oligonucleotides attached to magnetic beads. Following purification, the mRNA was fragmented using divalent cations at an elevated temperature. The mRNA fragments were converted to double-stranded cDNA (ds cDNA) using SuperScript II (Invitrogen, Carlsbad, CA, USA), RNase H, and DNA Pol I, primed by random primers. The ds cDNA was purified with AMPure XP beads (Agencourt, Beckman Coulter). The cDNA products were incubated with Klenow DNA polymerase to add an “A” base (adenine) to the 3′ end of the blunt DNA fragments. DNA fragments were ligated to Illumina unique dual index Y-adapters, which have a single “T” base (thymine) overhang at their 3′ ends. The adapter-ligated DNA products were purified with AMPure XP beads. Adapter-ligated DNA was amplified in a linker-mediated PCR (LM-PCR) for 10 cycles using Phusion DNA polymerase and Illumina’s PE genomic DNA primer set followed by purification with AMPure XP beads. Finally, the quality and quantity of the finished libraries were assessed using an Agilent DNA1000 chip (Agilent Technologies, Inc., Santa Clara, CA, USA) and Qubit dsDNA HS assay kit (Invitrogen, Carlsbad, CA, USA), respectively. Libraries were standardized to 2 nM. Paired-end 2× 150-bp sequencing was performed using standard SBS chemistry (v3) on an Illumina NovaSeq6000 sequencer. Images were analyzed using the standard Illumina Pipeline, version 1.8.2.

### Differential expression analysis.

Paired-end Illumina sequence read files were evaluated for quality and the absence of adaptor sequence using FastQC (https://www.bioinformatics.babraham.ac.uk/projects/fastqc/). Read files were mapped to C. albicans reference genome SC5314 (FungiDB), and gene transcript expression was quantified using HISAT2 and StringTie (54). Differential expression fold change, Wald test *P* values, and Benjamini-Hochberg adjustment for multiple comparisons were determined using DESeq2. Principal-component analysis was performed on regularized log-transformed gene counts to confirm the absence of batch effects (55).

### Spot dilution phenotypic assays.

The designated strains were grown overnight in YPD at 30°C, back diluted the next day into fresh YPD, and grown for 4 h at 30°C. The cells were further harvested and adjusted to an optical density at 600 nm (OD_600_) density of 0.1, and 10-fold serial dilutions were performed and spotted on either YPD or YPD with the designated concentration of calcofluor white, Congo red, or tunicamycin. The plates were then incubated at 30°C and pictures were taken after 2 and 3 days of incubation. The assays were conducted in duplicates or triplicates on different days to confirm reproducibility.

### Assays of hyphal morphogenesis.

For assays in liquid SM, strains were incubated overnight in YPD at 30°C, harvested, diluted into SM at 1:100 ratio, and incubated at 37°C for 4 h. Cells were collected and either fixed with formaldehyde or examined by light microscopy directly. For assays on solid media, cells were grown overnight in YPD at 30°C, harvested, and adjusted to an OD_600_ of 0.1. Tenfold serial dilutions were performed, and cells from the fourth dilution were collected and plated on the designated medium. For hypoxia phenotypes, the plates were incubated in a chamber with the designated amount of oxygen. For embedded experiments, cells were plated on the YPS medium, further overlaid with YPS top agar, and incubated at 25° or 37°C for 2 to 3 days.

### Microscopy.

For colony morphology analysis, plates were incubated for the designated time and imaged using a Nikon ES80 epifluorescence microscope equipped with a CoolSnap charge-coupled-device (CCD) camera at ×40 magnification. Live cell fluorescence imaging was carried out with a multiphoton laser scanning microscope (SP8; Leica Microsystems). Overnight cultures in YPD media were collected and back diluted either into fresh YPD or in the hyphal inducing medium to achieve either yeast or hyphal growth, respectively. Cells were then harvested, washed twice in 1× phosphate-buffered saline (PBS) and resuspended in 1× PBS prior to imaging. The samples were treated with Hoechst 33342 and incubated for 15 min in the dark at room temperature followed by washing twice with PBS. For Hoechst 33342 dye, an excitation wavelength of 350 nm and emission wavelength of 461 nm were used, whereas to visualize the GFP tag, an excitation wavelength of 395 nm and emission wavelength of 509 nm were used, and sequential images were acquired using 25× water immersive lens objective with a 3.32× zoom factor. Images were further analyzed by using ImageJ software.

### Transcriptional analysis by quantitative reverse transcription-PCR.

Strains were precultured overnight in YPD at 30°C, back diluted into either fresh YPD or in hypha-inducing medium, and collected after 4 to 5 h of incubation either at 30° or 37°C. RNA was isolated using a RiboPure kit and reverse transcribed using an iScript cDNA synthesis kit (170-8891; Bio-Rad). The qPCR was performed using IQ Sybr green supermix (170-8882; Bio-Rad), and primers used in this study are listed in Table S2. Briefly, each reaction contained 10 μl of the SYBR green PCR master mix, 0.10 μM the respective primers, and 150 ng of cDNA as a template in a total volume of 20 μl. Data analysis was performed using the comparative threshold cycle (2^−ΔΔ^*^CT^*) method, and *ACT1* was used as an internal control. Data reported here are the means from 3 independent biological replicates performed in triplicates.

### Data availability.

All experimental data are provided in the manuscript, supplemental files, or, for large gene expression data sets, deposited at the NCBI Gene Expression Omnibus site under accession number GSE155450.
