# Supplementary figures and images for: The Ndr/LATS Kinase Cbk1 Regulates a Specific Subset of Ace2 Functions and Suppresses the Hypha-to-Yeast Transition in Candida albicans
Source: mBio. 2020 Aug 18;11(4):e01900-20. doi: 10.1128/mBio.01900-20 (PMC7439482; doi:10.1128/mBio.01900-20)

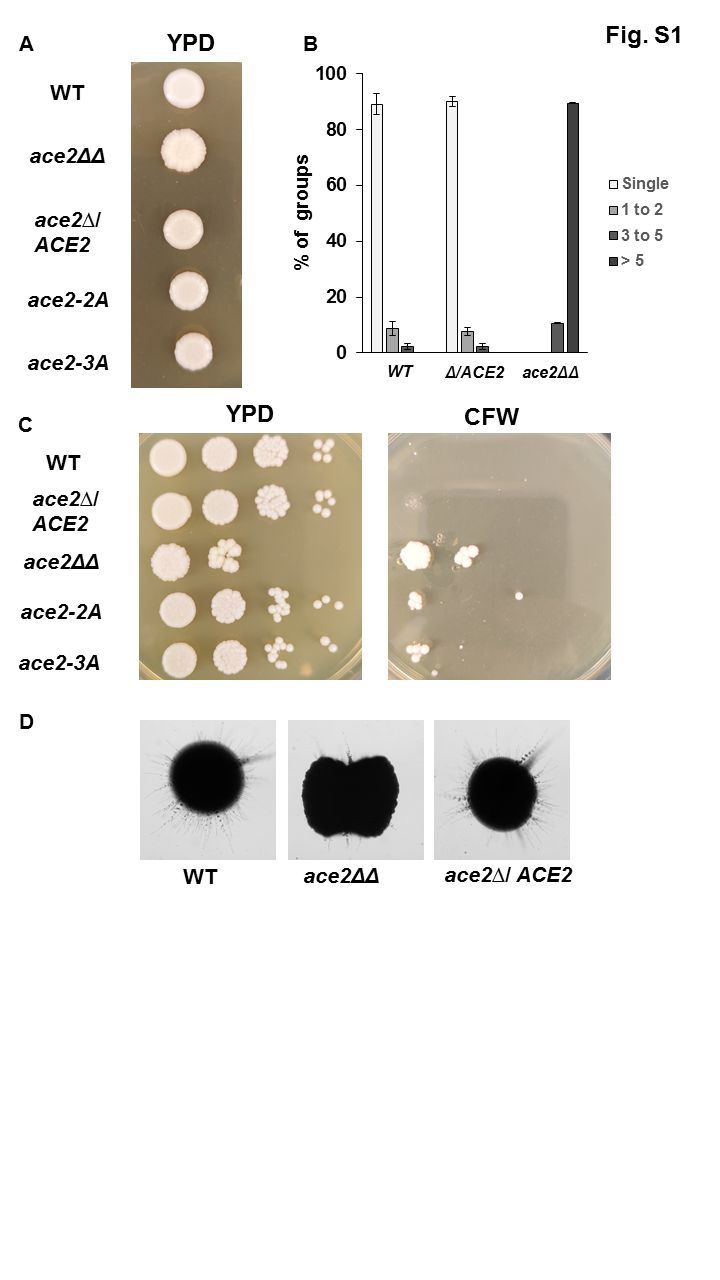

Supplement: FIG S1 [file mBio.01900-20-sf001.tif]

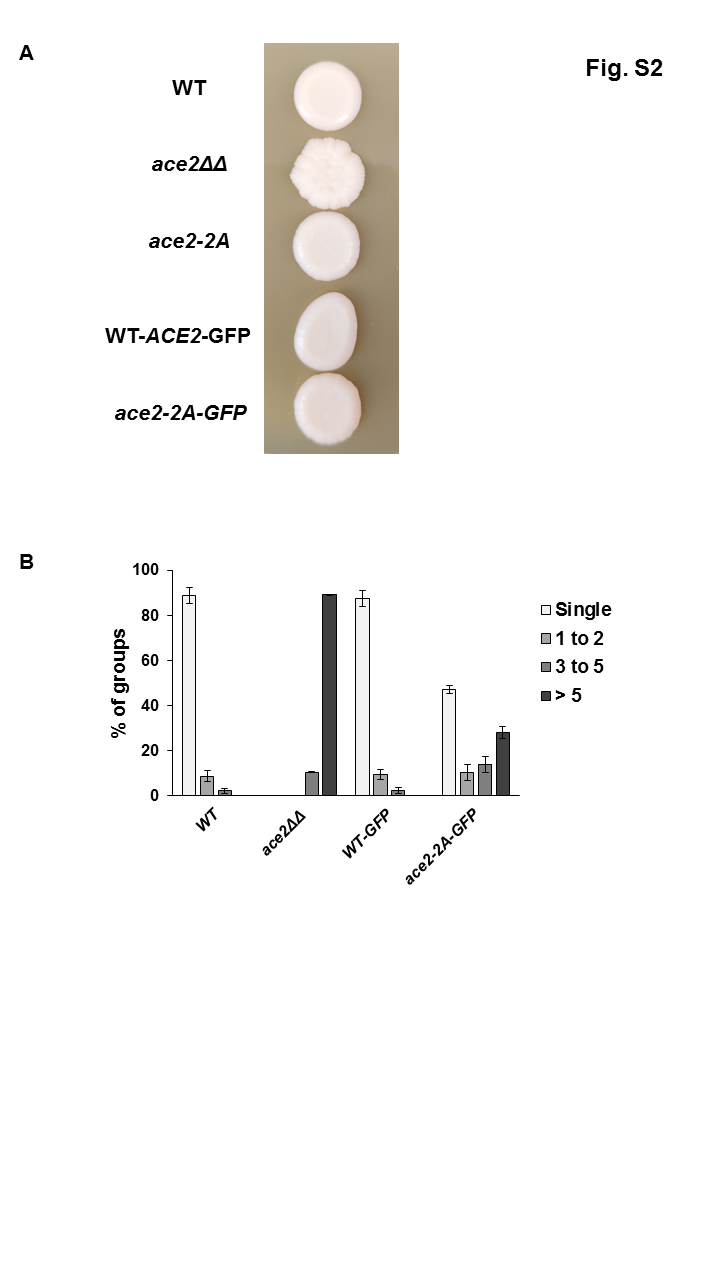

Supplement: FIG S2 [file mBio.01900-20-sf002.tif]
