# Supplementary material for: The Ndr/LATS Kinase Cbk1 Regulates a Specific Subset of Ace2 Functions and Suppresses the Hypha-to-Yeast Transition in Candida albicans
Source: mBio. 2020 Aug 18;11(4):e01900-20. doi: 10.1128/mBio.01900-20 (PMC7439482; doi:10.1128/mBio.01900-20)
Supplement: TABLE S1 [file mBio.01900-20-st001.docx]

**Table S1: Strain Table**

| **Strain** | **Genotype** |  |
| --- | --- | --- |
| SC5314 | Wild type clinical isolate | [1] |
| SN87 | *leu2*Δ/*leu2*Δ *his1*Δ/*his1*Δ *URA3*/*ura3*Δ::*imm 434* *IRO1/iro1Δ*::*imm 434* | [2] |
| SN152 | \| CAI4 with *iro1::IRO1/iro1*::*λ*imm434 *his*1::*hisG*/*his1*::*hisG leu2∆/leu2∆ arg4∆/arg4∆* \| \| --- \| | [2] |
| BWP17 | *ura3∆::λimm434 arg4::hisG his1::hisG*  *ura3∆::λimm434 arg4::hisG his1::hisG* | [3] |
| *snf5Δ/Δ* | As BWP17 with *snf5Δ :: ARG4/* *snf5Δ ::URA3* | [4] |
| *ace2Δ/ACE2* | As SN152 with *ace2Δ*::*LEU2*/*ACE2* | This Study |
| *ace2Δ/Δ*   \|  \| \| --- \| | As SN152 with *ace2Δ::LEU2/ace2Δ::LEU2* | This Study |
| *ace2-2A* | As SN152 with *ace2Δ::LEU2/ACE2::ace2^S136A, S151A^* ::*dpl200*::*ARG4* | This Study |
| *ace2-3A* | As SN152 with *ace2Δ::LEU2 /ace2^T49A^,^S136A, S151A^::dpl200*::*ARG4* | This Study |
| *ACE2-GFP* | As SN152 with *ACE2/ACE2-GFP::NAT1* | This Study |
| *ace2-2A- GFP* | As *ace2-2A* with *ace2Δ::LEU2/ ace2^S136A, S151A^-GFP::NAT1*::*dpl200*::*ARG4* | This Study |

1. Gillum, A.M., E.Y. Tsay, and D.R. Kirsch, *Isolation of the Candida albicans gene for orotidine-5'-phosphate decarboxylase by complementation of S. cerevisiae ura3 and E. coli pyrF mutations.* Mol Gen Genet, 1984. **198**(2): p. 179-82.

2. Noble, S.M. and A.D. Johnson, *Strains and strategies for large-scale gene deletion studies of the diploid human fungal pathogen Candida albicans.* Eukaryot Cell, 2005. **4**(2): p. 298-309.

3. Wilson, R.B., D. Davis, and A.P. Mitchell, *Rapid hypothesis testing with Candida albicans through gene disruption with short homology regions.* J Bacteriol, 1999. **181**(6): p. 1868-74.

4. Finkel, J.S., et al., *Portrait of Candida albicans adherence regulators.* PLoS Pathog, 2012. **8**(2): p. e1002525.
